# The Association of Fall Mechanism, Leukocyte Count, and Glucose Level With Undesirable Outcomes in Patients With Blunt Trauma Intracranial Hemorrhage: A Retrospective Study

**DOI:** 10.7759/cureus.109266

**Published:** 2026-05-20

**Authors:** C. Michael Dunham, Gregory S Huang

**Affiliations:** 1 Trauma, Critical Care, and General Surgery Services, St. Elizabeth Youngstown Hospital, Youngstown, USA

**Keywords:** adverse outcomes, fall mechanisms, glasgow coma scale, stress hyperglycemia, stress leukocytosis, trauma centers, traumatic intracranial hemorrhage

## Abstract

Objective

This study aimed to compare ground-level fall (GLF) with other mechanisms, admission white blood cell count (WBC), and blood glucose levels to determine their associations with multiple undesirable outcomes, including hospital death, increased ICU stay, increased hospital stay, adverse outcomes, not following commands at hospital discharge, and the need for mechanical ventilation.

Methods

Patients with intracranial hemorrhage (ICH) on CT, an admission Glasgow Coma Scale (GCS) score of 3-15, and blunt trauma were included. The brain CT Abbreviated Injury Scale score (ICH AIS) and Injury Severity Score (ISS) were obtained from the trauma registry. The extracranial (EC)-ISS was calculated as (the total ISS) - (the ICH AIS)^2^. An electronic medical record audit was performed to categorize injury mechanisms and retrieve WBC and glucose levels. An adverse outcome was defined as hospital death, ICU stay of ≥2 days, or hospital stay of >5 days. Hypotension was defined as an admission systolic blood pressure <100 mmHg. Hyperglycemia was defined as a glucose level ≥150 mg/dL. Leukocytosis was defined as a WBC ≥13.0/µL.

Results

The cohort consisted of 1,545 patients with ICH. The WBC and glucose values were available in 99% of patients. Non-GLF patients had higher proportions of age <70 years, GCS 3-12, ISS ≥16, hypotension, hyperglycemia, leukocytosis, death, ICU stay ≥2 days, hospital stay >5 days, an adverse outcome, not following commands at hospital discharge, and a need for mechanical ventilation (p≤0.01). Hyperglycemia had univariate associations with death, ICU stay ≥2 days, hospital stay >5 days, an adverse outcome, not following commands at hospital discharge, and a need for mechanical ventilation (p<0.01). Leukocytosis had univariate associations with death, ICU stay ≥2 days, hospital stay >5 days, an adverse outcome, not following commands at hospital discharge, and a need for mechanical ventilation (p<0.01). Hospital death showed an independent association with decreased GCS (p<0.01), increased ICH AIS, and hypotension (p=0.01). Increased ICU stay showed an independent association with decreased GCS (p<0.01), increased EC-ISS (p<0.01), increased ICH AIS (p<0.01), hyperglycemia (p<0.01), and leukocytosis (p<0.01). Increased hospital stay showed an independent association with decreased GCS (p<0.01), increased EC-ISS (p<0.01), increased ICH AIS (p<0.01), hyperglycemia (p<0.01), and leukocytosis (p<0.01). An adverse outcome showed an independent association with decreased GCS (p<0.01), increased EC-ISS (p<0.01), hypotension (p=0.01), increased ICH AIS (p<0.01), hyperglycemia (p<0.01), and leukocytosis (p<0.01). Not following commands at hospital discharge showed an independent association with decreased GCS (p<0.01) and increased ICH AIS (p<0.01). The need for mechanical ventilation showed an independent association with decreased GCS (p<0.01), increased EC-ISS (p<0.01), increased ICH AIS (p<0.01), hyperglycemia (p<0.01), and leukocytosis (p<0.01).

Conclusions

When compared with GLF patients, those with other blunt trauma mechanisms have worse risk profiles and poorer outcomes among patients with ICH. Stress leukocytosis and hyperglycemia were frequently found to be associated with multiple poor outcomes. Future investigators should consider incorporating stress leukocytosis and hyperglycemia as indicators of risk conditions that likely stem from catecholamine responses after trauma.

## Introduction

Several recent studies have demonstrated that patients experience substantial mortality, require hospitalization, or need intensive care when involved in a fall from a height [1-5], down the stairs [6,7], or at ground level [1,8-11]. Traumatic brain injury (TBI) is frequently encountered in patients who sustain a fall from a height [2,3,5], down the stairs [6,7], or at ground level [7,10,11].

We have found only one study in which all patients were considered to have sustained a TBI and were admitted to a hospital following a fall [1]. The patients were divided into two groups: low-energy falls and high-energy falls. Low-energy falls were defined as falls from ground level up to 3 feet, whereas high-energy falls were defined as those from heights greater than 3 feet. Patient risk conditions and undesirable outcomes were similar between the groups. The authors concluded that high-energy falls do not result in more severe injuries compared with low-energy falls [1]. In that study, TBI was identified according to Abbreviated Injury Scale (AIS) codes stored in the trauma registry. TBI severity was based on Glasgow Coma Scale (GCS) levels (mild, moderate, and severe), which were similar between the groups. It is notable that neither the AIS scores nor the brain CT results for the two groups were reported in the manuscript. The investigation also found that age was higher in the low-energy falls group than in the high-energy falls group [1]. This increased age may act as a confounding factor regarding coronary artery disease and the use of anticoagulants.

We have also found only one study that compared TBI patients with either a low-energy fall mechanism or a high-energy transfer mechanism (i.e., patients with other blunt trauma) [12]. Hospital mortality rates did not differ between the two groups. Of concern, most patients with brain injury had normal brain CT findings, and the majority were not admitted to the hospital.

In a subset of the current study population, we have shown that multiple undesirable outcomes (hospital death, increased ICU stay, increased hospital stay, an adverse outcome, not following commands at hospital discharge, and a need for mechanical ventilation) were associated with admission GCS, extracranial Injury Severity Score (EC-ISS), admission hypotension, head AIS, and admission glucose levels [13]. Several trauma studies have reported that risk conditions and undesirable outcomes are associated with elevated admission white blood cell (WBC) counts [14-18]. In particular, admitted trauma patients with increased neutrophil counts had lower GCS scores and increased ISS, hospital stay, and mortality [14]. Two other trauma studies have shown that leukocytosis is associated with major injury [15,16]. The ISS has been positively correlated with WBC values in one of these investigations [16]. In studies of patients with severe TBI, increased admission WBC values have been associated with unfavorable GCS outcomes at six months

The current study focuses on a cohort of blunt trauma patients with intracranial hemorrhage (ICH) and the need for admission to a level I trauma center. The first objective was to compare risk conditions (GCS, ISS, head AIS, EC-ISS, WBC levels, glucose values, and admission hypotension) and undesirable outcomes (hospital death, increased ICU stay, increased hospital stay, an adverse outcome, not following commands at hospital discharge, and a need for mechanical ventilation) according to the mechanism of injury. Specifically, ground-level falls (falls from a height <3 feet, from a bed, or while standing or sitting) were compared with other blunt trauma mechanisms (falls down multiple steps, non-ground-level falls, blunt assaults, motor vehicle crashes, motorcycle crashes, all-terrain vehicle crashes, pedestrians struck by a vehicle, or bicycle crashes). The second aim was to assess univariate and multivariate associations between risk conditions (GCS, ISS, head AIS, EC-ISS, WBC levels, glucose values, and admission hypotension) and undesirable outcomes (hospital death, ICU stay, hospital stay, adverse outcomes, command-following status at discharge, and the need for mechanical ventilation) irrespective of the mechanism of injury. The final objective was to determine whether ISS or the mechanism of injury contributed to the multivariate prediction of undesirable outcomes.

## Materials and methods

Ethics statement

This study was reviewed by the local institutional review board (IRB) and found to qualify for exemption under Exempt Category 4 (Bon Secours Mercy Health, IRB number 2026-Trauma-Dunham3). The need for informed consent was waived because of minimal risk and the retrospective nature of the investigation. All medical records were de-identified.

Study design and population

In this retrospective study, the parent group data source consisted of adult blunt trauma patients with ICH admitted to a level I trauma center in Northeast Ohio. The time periods included January 21 to July 21 in 2018, 2019, and 2020, and January 1 to December 31 in 2021, 2022, 2023, and 2024. The partial years for 2018-2020 were derived from a database previously used in a publication addressing the Ohio stay-at-home order for COVID-19 [19].

Inclusion and exclusion criteria

Data obtained from the trauma registry were used to identify the patient cohort of trauma center admissions, including patients aged ≥18 years with blunt trauma and ICH. Exclusion criteria included patients younger than 18 years, those with a non-blunt trauma mechanism, patients without ICH, or those not admitted to the trauma center.

Data collection

Trauma Registry

Data from the trauma registry included age, GCS [20], ISS [21], highest head AIS [22], hospital mortality, admission systolic blood pressure, ICU stay (days), hospital stay (days), and need for mechanical ventilation. Because a trauma registry audit demonstrated that the highest head AIS was directly related to the magnitude of the ICH in >90% of the patients, the highest head AIS is subsequently referred to as ICH AIS. The EC-ISS was computed as follows: (the total ISS) - (the ICH AIS)^2^; this computation has been shown to have univariate and multivariate associations with undesirable outcomes [13].

Electronic Medical Records

All patients dying in the hospital were classified as not following commands at hospital discharge. For hospital survivors, the electronic medical records were reviewed to classify patients as either following or not following commands at discharge. The electronic medical records of all patients were also reviewed to capture a blood glucose level obtained within eight hours of hospital admission. Similarly, the records were reviewed to capture a WBC level obtained within eight hours of admission. An admission WBC was considered undocumented if a value could not be found, the value was <4.0/µL with active chemotherapy, or the value was >30,000/µL with a diagnosis of leukemia.

Risk conditions and undesirable or poor outcomes

Risk condition considerations were trauma mechanism (ground-level fall or other blunt trauma mechanism), decreased GCS, increased ICH AIS, increased ISS, increased EC-ISS, systolic blood pressure <100 mmHg (hypotension), glucose level ≥150 mg/dL (hyperglycemia), and WBC ≥13.0/µL (leukocytosis). Using data from the trauma registry and electronic medical records, six undesirable or poor outcomes were delineated: hospital death, an ICU stay of ≥2 days, a hospital stay of >5 days, an adverse outcome, failure to follow commands at hospital discharge, and a need for mechanical ventilation. An adverse outcome was considered to be present if a patient died in the hospital, had an ICU stay of ≥2 days, or had a hospital stay of >5 days.

Mechanisms of injury classification

Most patients in the 2018-2020 cohort were classified by the trauma registrars as having a fall mechanism of injury. An electronic medical record review was undertaken to further classify these patients as having either a ground-level fall (from a height <3 feet, or from a bed, or while standing or sitting) or other fall mechanism (a fall down ≥4 steps or from a height ≥3 feet). An audit showed that these two groups had significantly different risk profiles and outcomes. Accordingly, patients with a fall down multiple steps or from a height were reclassified into the other blunt trauma mechanisms group. Using this method, the 2021-2024 cohort underwent electronic medical record review to classify each patient as having a ground-level fall or another blunt trauma mechanism. Ground-level falls included those from a standing height, chair, commode, or bed. The other blunt trauma mechanism group included falls down multiple steps, non-ground-level falls, blunt assaults, motor vehicle crashes, motorcycle crashes, all-terrain vehicle crashes, pedestrians struck by a vehicle, or bicycle crashes.

Risk score for an adverse outcome

Based on the univariate associations of risk conditions with adverse outcomes, a risk score was created. The risk score was the sum of GCS 3-8 (0/1), EC-ISS ≥6 (0/1), ICH AIS ≥4 (0/1), hypotension (0/1), hyperglycemia (0/1), and leukocytosis (0/1), ranging from 0 to 6. The relationship between the risk score and the presence of an adverse outcome was assessed using receiver operating characteristic (ROC) curve analysis. This analysis identified a cutoff point for a dichotomous assessment to quantify the score's potential for risk stratification.

Multivariate assessment of risk conditions for undesirable outcomes

A multivariate assessment of the six dependent undesirable outcome models (an adverse outcome, death, increased ICU days, increased hospital stay, not following commands at hospital discharge, and a need for mechanical ventilation) was undertaken for all patients without regard for mechanisms of injury. For each undesirable outcome model, multivariate p-values were assessed for each of the six potential independent variables (decreased GCS, increased EC-ISS, hypotension, increased ICH AIS, hyperglycemia, and leukocytosis). All multivariate information was available for 1,530 (99.0%) patients. A non-fall mechanism (yes or no) and separately an ISS ≥16 (yes or no) were added to the other six potential independent variables to assess the increment in the R-squared value of each model.

Statistical analysis

Continuous data without a normal distribution (e.g., age, WBC values, and glucose levels) and ordinal data (e.g., ISS, GCS, and ICH AIS) are presented as the median and interquartile range (IQR) or as a proportion (e.g., ISS ≥16, EC-ISS ≥6, hypotension, hyperglycemia, and leukocytosis). Proportional data are presented as the count (N) and percent.

For dichotomous proportional data conforming to a 2 x 2 contingency table format (e.g., age ≥70 years, GCS 3-8, GCS 3-12, ISS ≥16, EC-ISS ≥6, hypotension, hyperglycemia, and leukocytosis, a ground-level fall mechanism, an adverse outcome, a hospital death, an ICU stay ≥2 days, a hospital stay >5 days, not following commands at hospital discharge, and a need for mechanical ventilation), a chi-square test was used for analysis. The odds ratio (OR) and risk ratio (RR) were calculated to quantify proportional differences. When comparing the median values between two independent groups, the Wilcoxon rank sum test p-value was used (e.g., ISS, WBC, age, and glucose).

The Spearman correlation coefficient was computed for the relationship between WBC and glucose values. A risk score cutoff point for an adverse outcome was assessed using ROC curve analyses. Multivariate regression analysis was used to assess simultaneous associations of independent variables with a dependent variable. Multivariate logistic regression analysis was used to analyze data with a dichotomous dependent variable (mortality, adverse outcome, no hospital discharge commands, and a need for mechanical ventilation). Multivariate linear regression analysis was used for data with a continuous dependent variable (ICU and hospital stays (days)).

Data were entered into Excel 2010 (Microsoft Corp., Redmond, WA) and imported into SAS System for Windows (release 9.2; SAS Institute Inc., Cary, NC). For ROC curve analyses, data were exported from SAS into MedCalc® Statistical Software (version 22.016; MedCalc Software Ltd., Ostend, Belgium). The significance level for the p-value was set at <0.05.

## Results

Audit of patients with a fall mechanism of injury (2018-2020)

Of the 436 blunt trauma patients with ICH, 345 (79.1%) were classified by the trauma registrars as having a fall mechanism of injury. An electronic medical record audit was performed by the first author to classify each fall patient as having a ground-level fall, a fall down multiple steps, or a fall from a height. Table 1 presents a comparison of ground-level falls with falls down multiple steps or from a height. Compared with ground-level falls, falls down multiple steps or from a height were associated with a lower proportion of patients aged ≥70 years and higher proportions of GCS 3-8, GCS 3-12, EC-ISS ≥6, leukocytosis, hospital stay >5 days, and need for mechanical ventilation.

**Table 1:** Analysis by fall mechanism (2018-2020) GCS: Glasgow Coma Scale score; ICH: intracranial hemorrhage; AIS: Abbreviated Injury Scale score; ISS: Injury Severity Score; EC: extracranial; ICU: intensive care unit; LOS: length of stay

Variable	Ground-level fall, n (%)	Fall down multiple Steps/from a height, n (%)	χ^2^ p-Value	χ^2^ value
Number of patients (%)	292 (84.6%)	53 (15.4%)	–	–
Age ≥70 years	220 (75.3%)	21 (39.6%)	<0.0001	27.2
GCS 3-8	13 (4.5%)	7 (13.2%)	0.0121	6.3
GCS 3-12	24 (8.2%)	9 (17.0%)	0.0450	4.0
ICH AIS 4-5	150 (51.4%)	23 (43.4%)	0.2855	1.1
ISS ≥16	152 (52.1%)	23 (54.7%)	0.7211	0.1
EC-ISS ≥6	16 (5.5%)	13 (24.5%)	<0.0001	21.1
Leukocytosis	47/290 (16.2%)	17/53 (32.1%)	0.0064	7.4
Mortality	22 (7.5%)	3 (5.7%)	0.6283	0.2
ICU stay ≥2 days	67 (23.0%)	18 (34.0%)	0.0868	2.9
LOS >5 days	83 (28.4%)	24 (45.3%)	0.0146	6.0
Adverse outcome	111 (38.0%)	25 (47.2%)	0.2095	1.6
Ventilator	29 (9.9%)	14 (26.4%)	0.0008	11.2

Analyses were undertaken to compare the 91 patients classified by the trauma registrars as having a non-fall blunt trauma mechanism with the 53 patients classified by the current investigators as having a fall down ≥4 steps or from a height >3 feet. The proportions of patients with GCS 3-8 were similar for the fall (13.2%) and non-fall (25.3%; p=0.0855) groups. The proportions of patients with ICH AIS 4-5 were similar for the fall (43.4%) and non-fall (52.8%; p=0.2791) groups. The proportions of patients with ISS ≥16 were similar for the fall (54.7%) and non-fall (63.9%; p=0.1815) groups. The proportions of patients requiring mechanical ventilation were similar for the fall (26.4%) and non-fall (41.8%; p=0.0645) groups.

Categorization by mechanism of injury (2018-2024)

An electronic medical record audit was performed to determine the mechanism of injury for each patient (n=1,545), with patients categorized as having either a ground-level fall or another blunt trauma mechanism. Table 2 shows a comparison of risks and outcomes between patients with a ground-level fall and those with another blunt trauma mechanism. Compared with patients with ground-level falls, those with another blunt trauma mechanism had a lower proportion of patients aged ≥70 years and higher proportions of GCS 3-8, GCS 3-12, ISS ≥16, EC-ISS ≥6, hypotension, hyperglycemia, and leukocytosis. Patients with another blunt trauma mechanism also had higher proportions of mortality, failure to follow commands at hospital discharge, an ICU stay of ≥2 days, a hospital stay of >5 days, adverse outcomes, and a need for mechanical ventilation. Patients with ground-level falls accounted for 53.6% (397/741) of those with an adverse outcome and had a total hospital stay of 4,894 days, equivalent to 889.8 days per year over the 5.5-year study period. Of the 811 ground-level fall survivors, 457 (52.1%) were discharged to acute rehabilitation, subacute nursing care, or a long-term acute care facility.

**Table 2:** Blunt trauma patients with ICH during 2018-2024 (n=1,545) ^╠^A few values were missing GLF: ground-level fall; χ2: chi-square; OR: odds ratio; RR: risk ratio; IQR: interquartile range; GCS: Glasgow Coma Scale score; ICH: intracranial hemorrhage; AIS: Abbreviated Injury Scale score; ISS: Injury Severity Score; EC: extracranial; DC: discharge; ICU: intensive care unit; LOS: hospital length of stay

Variable	GLF	Other mechanisms	χ^2^ p-Value	χ^2^ value	OR	RR	Wilcoxon p-Value
Number of patients (%)	947 (61.3%)	598 (38.7%)	–	–	–	–	–
Age ≥70 years, n (%)	679 (71.7%)	142 (23.8%)	<0.0001	338.5	8.1	3.0	–
Age, years, median (IQR)	77 (68-85)	55 (36-68)	–	–	–	–	<0.0001
GCS 3-8, n (%)	60 (6.3%)	139 (23.2%)	<0.0001	93.4	0.22	0.27	–
GCS 3-12, n (%)	98 (10.4%)	180 (30.1%)	<0.0001	96.9	0.27	0.34	–
ICH AIS ≥4, n (%)	374 (39.5%)	266 (44.5%)	0.0525	3.8	–	–	–
ISS ≥16, n (%)	396 (41.8%)	355 (59.4%)	<0.0001	45.23	0.49	0.70	–
EC-ISS ≥6, n (%)	80 (8.5%)	227 (38.0%)	<0.0001	200.5	0.15	0.22	–
Hypotension, n (%)	38 (4.0%)	45 (7.5%)	0.0029	8.9	0.51	0.53	–
Death, n (%)	66 (7.0%)	68 (11.4%)	0.0027	9.0	0.58	0.61	–
ICU stay ≥2 days, n (%)	234 (24.7%)	284 (47.5%)	<0.0001	85.4	0.36	0.52	–
LOS >5 days, n (%)	298 (31.5%)	263 (44.0%)	<0.0001	24.8	0.59	0.72	–
LOS, days, median (IQR)	4 (2-6)	5 (2-10)	–	–	–	–	<0.0001
Adverse outcome	397 (41.9%)	344 (57.5%)	<0.0001	35.8	0.53	0.73	–
Not following DC commands, n (%)	79 (8.3%)	105 (17.6%)	<0.0001	30.0	0.43	0.48	–
Ventilator, n (%)	93 (9.8%)	184 (30.8%)	<0.0001	109.3	0.25	0.32	–
Hyperglycemia^╠^, n (%)	251/945 (26.6%)	193/594 (32.5%)	0.0124	6.3	0.75	0.82	–
Leukocytosis^╠^, n (%)	167/939 (17.8%)	260/592 (43.9%)	<0.0001	123.3	0.28	0.40	

An admission WBC was not documented in 14 (0.9%) patients because the value could not be found, was <4.0/µL in the setting of active chemotherapy, or was >30,000/µL in patients with a diagnosis of leukemia. The admission glucose level was missing in six (0.4%) patients. Both WBC and glucose levels exhibited a non-normal distribution (Shapiro-Wilk test, p<0.05). Table 3 presents various WBC and glucose level cutoff points for patients without and with an adverse outcome across all trauma mechanisms. A WBC ≥13.0/µL was observed in 27.9% of patients (427/1,531), and a glucose level ≥150 mg/dL was observed in 28.9% (444/1,539). These cutoff points approximate the upper 25th percentile of WBC and glucose levels for the total population and were therefore selected as discriminant thresholds for leukocytosis and hyperglycemia.

**Table 3:** White blood cell and glucose cutoff points for those without and with an adverse outcome Adverse outcomes were defined as hospital death, an ICU stay of ≥2 days, or a hospital stay of >5 days χ2: chi-square; OR: odds ratio; RR: risk ratio; WBC: white blood cell count; IQR: interquartile range

Variable	No adverse outcome	Adverse outcome	χ^2 ^p-Value	χ^2^ value	OR	RR	Wilcoxon p-Value
WBC ≥12.5/µL, n (%)	132/795 (16.6%)	348/736 (47.3%)	<0.0001	167.1	4.5	2.8	–
WBC ≥13.0/µL, n (%)	104/795 (13.1%)	323/736 (43.9%)	<0.0001	180.3	5.2	3.4	–
WBC ≥13.5/µL, n (%)	89/795 (11.2%)	291/736 (39.5%)	<0.0001	164.5	5.2	3.5	–
WBC ≥14/µL, n (%)	79/795 (9.9%)	262/736 (35.6%)	<0.0001	145.4	5.0	3.6	–
Median WBC, median (IQR)	9.0 (7.0-11.3)	12.2 (8.9-15.0)	–	–	–	–	<0.0001
Glucose level ≥145 mg/dL, n (%)	162/801 (20.2%)	330/738 (44.7%)	<0.0001	105.9	3.2	2.2	–
Glucose level ≥150 mg/dL, n (%)	142/801 (17.7%)	302/738 (40.9%)	<0.0001	100.7	3.2	2.3	–
Glucose level ≥155 mg/dL, n (%)	127/801 (15.9%)	270/738 (36.6%)	<0.0001	86.2	3.1	2.3	–
Glucose level ≥160 mg/dL, n (%)	108/801 (13.5%)	248/738 (33.6%)	<0.0001	87.5	3.2	2.5	–
Median glucose level, median (IQR)	115.0 (101-138)	139.0 (115-174)	–	–	–	–	<0.0001

Table 4 presents the risk conditions associated with hyperglycemia and leukocytosis across all trauma mechanisms. Although hyperglycemia did not vary by age, its proportions were higher in patients with other blunt trauma mechanisms, GCS 3-8, ISS ≥16, EC-ISS ≥6, ICH AIS 4-5, and hypotension compared with their counterparts. Proportions of leukocytosis were higher in patients aged <70 years, those with other blunt trauma mechanisms, GCS 3-8, ISS ≥16, EC-ISS ≥6, ICH AIS 4-5, and hypotension compared with their counterparts. WBC and glucose levels showed a significant positive correlation (p<0.0001; r=0.24). Patients with hyperglycemia had a higher proportion of leukocytosis (42.9%; 189/441) than patients without hyperglycemia (21.8%; 237/1,089; p<0.0001; OR=2.7; RR=2.0).

**Table 4:** Risk conditions for increased glucose values and increased white blood cell counts among patients with blunt trauma ICH The glucose level was missing in six patients; the white blood cell count was missing in 14 patients χ2: chi-square; GCS: Glasgow Coma Scale score; ISS: Injury Severity Score; EC: extracranial; ICH: intracranial hemorrhage; AIS: Abbreviated Injury Scale score

Variable	Hyperglycemia, n (%)	χ^2^ p-Value	χ^2^ value	Leukocytosis, n (%)	χ^2^ p-Value	χ^2^ value
Age <70 years	206/721 (28.6%)	–	–	247/718 (34.4%)	–	–
Age ≥70 years	238/818 (29.1%)	0.8209	0.1	180/813 (22.1%)	<0.0001	28.5
Ground-level falls	251/945 (26.6%)	–	–	167/939 (17.8%)	–	–
Other mechanisms	193/594 (32.5%)	0.0124	6.3	260/592 (43.9%)	<0.0001	123.3
GCS 3-8	102/190 (51.5%)	–	–	125/196 (63.8%)	–	–
GCS 9-15	342/1341 (25.5%)	<0.0001	56.9	302/1335 (22.6%)	<0.0001	143.9
ISS ≥16	282/749 (37.7%)	–	–	291/747 (39.0%)	–	–
ISS <16	162/790 (20.5%)	<0.0001	55.1	136/784 (17.4%)	<0.0001	88.8
EC-ISS ≥6	139/305 (45.6%)	–	–	157/305 (51.5%)	–	–
EC-ISS <6	305/1234 (24.7%)	<0.0001	51.8	270/1226 (22.0%)	<0.0001	105.4
ICH AIS 4-5	225/638 (35.3%)	–	–	232/636 (36.5%)	–	–
ICH AIS <4	219/901 (24.3%)	<0.0001	21.9	195/895 (21.8%)	<0.0001	39.9
Hypotension	36/82 (43.9%)	–	–	40/81 (49.4%)	–	–
No hypotension	408/1457 (28.0%)	0.0020	9.6	387/1450 (26.7%)	<0.0001	19.6

Table 5 presents univariate associations between risk conditions and adverse outcomes for all trauma mechanisms. Patients with an adverse outcome had higher proportions of GCS 3-8, GCS 3-12, ISS ≥16, EC-ISS ≥6, ICH AIS ≥4, hypotension, hyperglycemia, and leukocytosis than patients without an adverse outcome.

**Table 5:** Risk conditions for an adverse outcome among patients with blunt trauma ICH ^╠^A few values were missing χ2: chi-square; OR: odds ratio; RR: risk ratio; GCS: Glasgow Coma Scale score; ISS: Injury Severity Score; EC: extracranial; ICH: intracranial hemorrhage; AIS: Abbreviated Injury Scale score

Variable	No adverse outcome, n (%)	Adverse outcome, n (%)	χ^2^ p-Value	χ^2^ value	OR	RR
Number of patients (%)	804 (52.0%)	741 (48.0%)	–	–	–	–
GCS 3-8	11 (1.4%)	188 (25.4%)	<0.0001	198.0	24.5	18.5
GCS 3-12	22 (2.7%)	256 (34.6%)	<0.0001	264.5	18.8	12.6
ISS ≥16	218 (27.1%)	533 (71.9%)	<0.0001	310.0	6.9	2.7
EC-ISS ≥6	75 (9.3%)	232 (31.3%)	<0.0001	117.0	4.4	3.4
ICH AIS ≥4	191 (23.8%)	449 (60.6%)	<0.0001	215.7	4.9	2.6
Hypotension	19 (2.4%)	64 (8.6%)	<0.0001	30.0	4.0	3.7
Hyperglycemia^╠^	142/801 (17.7%)	302/738 (40.9%)	<0.0001	100.7	3.2	2.3
Leukocytosis^╠^	104/795 (13.1%)	323/736 (43.9%)	<0.0001	180.3	5.2	3.4

Relationship between risk score and adverse outcome

**Figure 1:**
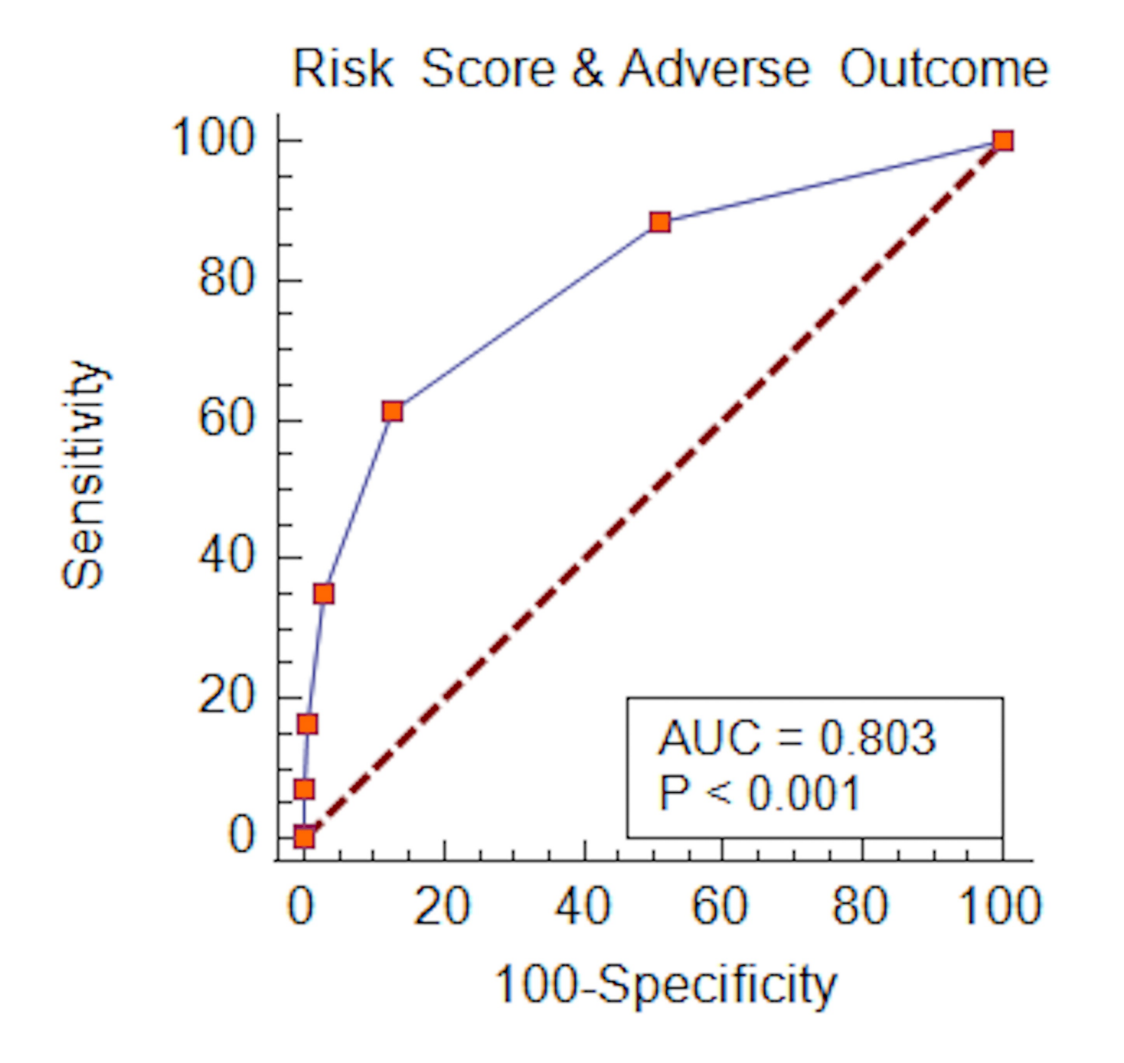
Relationship between the risk score and an adverse outcome AUC: area under the receiver operating characteristic curve

Figure 1 displays the ROC curve depicting the relationship between the risk score and adverse outcomes. The area under the curve (AUC) was 0.803 (95% confidence interval (CI)=0.782-0.822). A risk score of ≥2 was identified by ROC curve analysis as the optimal cutoff for distinguishing patients with and without an adverse outcome. The proportion of adverse outcomes was higher in patients with a risk score of 2-6 (81.6%; 453/555) compared with those with a risk score of 0-1 (28.9%; 282/975; χ² p<0.0001; χ²=393.5; OR=10.9; RR=2.8).

Table 6 shows univariate associations between risk conditions and mortality for all trauma mechanisms. Patients who died had higher proportions of GCS 3-8, GCS 3-12, ISS ≥16, EC-ISS ≥6, ICH AIS ≥4, hypotension, hyperglycemia, and leukocytosis than survivors.

**Table 6:** Risk conditions for death among patients with blunt trauma ICH ^╠^A few values were missing χ2: chi-square; OR: odds ratio; RR: risk ratio; GCS: Glasgow Coma Scale score; ISS: Injury Severity Score; EC: extracranial; ICH: intracranial hemorrhage; AIS: Abbreviated Injury Scale score

Variable	Alive, n (%)	Died, n (%)	χ^2^ p-Value	χ^2^ value	OR	RR
Number of patients (%)	1411 (91.3%)	134 (8.7%)	–	–	–	–
GCS 3-8	120 (8.5%)	79 (59.0%)	<0.0001	277.6	15.5	6.9
GCS 3-12	182 (12.9%)	96 (71.6%)	<0.0001	286.2	17.1	5.6
ISS ≥16	631 (44.7%)	120 (89.6%)	<0.0001	98.5	10.6	2.0
EC-ISS ≥6	259 (18.4%)	48 (35.8%)	<0.0001	23.5	2.5	2.0
ICH AIS ≥4	531 (37.6%)	109 (81.3%)	<0.0001	96.4	7.2	2.2
Hypotension	62 (4.4%)	21 (15.7%)	<0.0001	30.6	4.0	3.6
Hyperglycemia^╠^	377/1407 (26.8%)	67/132 (50.8%)	<0.0001	33.8	2.8	1.9
Leukocytosis^╠^	353/1400 (25.2%)	74/131 (56.5%)	<0.0001	58.3	3.9	2.2

Table 7 presents univariate associations between risk conditions and an ICU stay ≥2 days for all trauma mechanisms. Patients with an ICU stay of ≥2 days had higher proportions of GCS 3-8, GCS 3-12, ISS ≥16, EC-ISS ≥6, ICH AIS ≥4, hypotension, hyperglycemia, and leukocytosis than patients with an ICU stay of <2 days.

**Table 7:** Risk conditions for increased ICU stay among patients with blunt trauma ICH ^╠^A few values were missing ICU: intensive care unit; χ2: chi-square; OR: odds ratio; RR: risk ratio; GCS: Glasgow Coma Scale score; ISS: Injury Severity Score; EC: extracranial; ICH: intracranial hemorrhage; AIS: Abbreviated Injury Scale score

Variable	ICU stay <2 days, n (%)	ICU stay ≥2 days, n (%)	χ^2^ p-Value	χ^2^ value	OR	RR
Number	1027 (66.5%)	518 (33.5%)	–	–	–	–
GCS 3-8	41 (4.0%)	158 (30.5%)	<0.0001	215.7	10.6	7.6
GCS 3-12	63 (6.1%)	215 (41.5%)	<0.0001	292.0	10.9	6.8
ISS ≥16	329 (32.0%)	422 (81.5%)	<0.0001	336.8	9.3	2.5
EC-ISS ≥6	118 (11.5%)	189 (36.5%)	<0.0001	135.1	4.4	3.2
ICH AIS ≥4	287 (28.0%)	353 (68.2%)	<0.0001	229.3	5.5	2.4
Hypotension	37 (3.6%)	46 (8.9%)	<0.0001	18.9	2.6	2.5
Hyperglycemia^╠^	219/1022 (21.4%)	225/517 (43.5%)	<0.0001	81.6	2.8	2.0
Leukocytosis^╠^	168/1014 (16.6%)	259/517 (50.1%)	<0.0001	191.4	5.1	3.0

Table 8 displays univariate associations between risk conditions and a hospital stay of >5 days for all trauma mechanisms. Patients with a hospital stay of >5 days had higher proportions of GCS 3-8, GCS 3-12, ISS ≥16, EC-ISS ≥6, ICH AIS ≥4, hypotension, hyperglycemia, and leukocytosis than patients with a hospital stay of ≤5 days.

**Table 8:** Risk conditions for increased hospital stay among patients with blunt trauma ICH ^╠^A few values were missing LOS: hospital length of stay; χ2: chi-square; OR: odds ratio; RR: risk ratio; GCS: Glasgow Coma Scale score; ISS: Injury Severity Score; EC: extracranial; ICH: intracranial hemorrhage; AIS: Abbreviated Injury Scale score

Variable	LOS ≤5 days, n (%)	LOS >5 days, n (%)	χ^2^ p-Value	χ^2^ value	OR	RR
Number of patients (%)	984 (63.7%)	561 (36.3%)	–	–	–	–
GCS 3-8	69 (7.0%)	130 (23.2%)	<0.0001	83.2	4.0	3.3
GCS 3-12	104 (10.6%)	174 (31.0%)	<0.0001	101.2	3.8	2.9
ISS ≥16	349 (35.5%)	402 (71.7%)	<0.0001	187.3	4.6	2.0
EC-ISS ≥6	114 (11.6%)	193 (34.4%)	<0.0001	116.8	4.0	3.0
ICH AIS ≥4	314 (31.9%)	326 (58.1%)	<0.0001	101.1	3.0	1.8
Hypotension	40 (4.1%)	43 (7.7%)	0.0025	9.1	2.0	1.9
Hyperglycemia^╠^	222/980 (22.7%)	222/559 (39.7%)	<0.0001	50.5	2.2	1.8
Leukocytosis^╠^	182/972 (18.7%)	245/559 (43.8%)	<0.0001	111.2	3.4	2.3

Table 9 presents univariate associations between risk conditions and not following commands at hospital discharge for all trauma mechanisms. Patients who did not follow commands at hospital discharge had higher proportions of GCS 3-8, GCS 3-12, ISS ≥16, EC-ISS ≥6, ICH AIS ≥4, hypotension, hyperglycemia, and leukocytosis than patients who followed commands.

**Table 9:** Risk conditions for not following commands at hospital discharge among patients with blunt trauma ICH ^╠^A few values were missing χ2: chi-square; OR: odds ratio; RR: risk ratio; GCS: Glasgow Coma Scale score; ISS: Injury Severity Score; EC: extracranial; ICH: intracranial hemorrhage; AIS: Abbreviated Injury Scale score

Variable	Following commands, n (%)	Not following commands, n (%)	χ^2^ p-Value	χ^2^ value	OR	RR
Number of patients (%)	1361 (88.1%)	184 (11.9%)	–	–	–	–
GCS 3-8	76 (5.6%)	123 (66.9%)	<0.0001	532.1	34.1	12.0
GCS 3-12	132 (9.7%)	146 (79.4%)	<0.0001	526.6	35.8	8.2
ISS ≥16	583 (42.8%)	168 (91.3%)	<0.0001	152.4	14.0	2.1
EC-ISS ≥6	234 (17.2%)	73 (39.7%)	<0.0001	51.5	3.2	2.3
ICH AIS ≥4	486 (35.7%)	154 (83.7%)	<0.0001	153.8	9.2	2.3
Hypotension	58 (4.3%)	25 (13.6%)	<0.0001	27.7	3.5	3.2
Hyperglycemia^╠^	350/1357 (25.8%)	94/182 (51.7%)	<0.0001	52.3	3.1	2.0
Leukocytosis^╠^	325/1350 (24.1%)	102/181 (56.4%)	<0.0001	82.7	4.1	2.4

Table 10 shows univariate associations between risk conditions and the need for mechanical ventilation for all trauma mechanisms. Patients who required mechanical ventilation had higher proportions of GCS 3-8, GCS 3-12, ISS ≥16, EC-ISS ≥6, ICH AIS ≥4, hypotension, hyperglycemia, and leukocytosis than patients who did not require mechanical ventilation.

**Table 10:** Risk conditions for mechanical ventilation among patients with blunt trauma ICH ^╠^A few values were missing χ2: chi-square; OR: odds ratio; RR: risk ratio; GCS: Glasgow Coma Scale score; ISS: Injury Severity Score; EC: extracranial; ICH: intracranial hemorrhage; AIS: Abbreviated Injury Scale score

Variable	No ventilator, n (%)	Ventilator, n (%)	χ^2^ p-Value	χ^2^ value	OR	RR
Number of patients (%)	1268 (82.1%)	277 (17.9%)	–	–	–	–
GCS 3-8	50 (4.0%)	149 (53.8%)	<0.0001	503.4	28.4	13.7
GCS 3-12	92 (7.3%)	186 (67.2%)	<0.0001	552.7	26.1	9.4
ISS ≥16	501 (39.5%)	250 (90.3%)	<0.0001	234.3	14.2	2.3
EC-ISS ≥6	184 (14.5%)	123 (44.4%)	<0.0001	127.6	4.7	3.1
ICH AIS ≥4	432 (34.1%)	208 (75.1%)	<0.0001	157.7	5.8	2.2
Hypotension	52 (4.1%)	31 (11.2%)	<0.0001	22.5	3.0	2.7
Hyperglycemia^╠^	298/1263 (23.6%)	146/276 (52.9%)	<0.0001	94.8	3.6	2.2
Leukocytosis^╠^	263/1255 (21.0%)	164/276 (59.4%)	<0.0001	166.4	5.5	3.0

Table 11 displays the six dependent undesirable outcome models (an adverse outcome, death, an increased ICU stay, an increased hospital stay, not following commands at hospital discharge, and a need for mechanical ventilation) for all patients without regard for mechanisms of injury. For each undesirable outcome model, multivariate p-values are displayed for each of the six potential independent variables (decreased GCS, increased EC-ISS, hypotension, increased ICH AIS, hyperglycemia, and leukocytosis). Complete multivariate data were available for 1,530 (99.0%) patients. Hyperglycemia and leukocytosis were independently significant in four of the six models (adverse outcome, increased ICU stay, increased hospital stay, and need for mechanical ventilation). When other blunt trauma mechanisms (yes/no) or ISS ≥16 (yes/no) were added separately to the six independent variables, the increase in the R-squared value of each model was negligible (≤0.02).

**Table 11:** Multivariate risk condition summary for patients with blunt trauma ICH GCS: Glasgow Coma Scale score; EC: extracranial; ISS: Injury Severity Score; ICH: intracranial hemorrhage; AIS: Abbreviated Injury Scale score; GLF: ground-level fall; ICU: intensive care unit; LOS: hospital length of stay; DC: discharge

Variables	Decreased GCS	Increased EC-ISS	Hypotension	Increased ICH AIS	Hyperglycemia	Leukocytosis	Six-item R-squared value	GLF-added R-squared value	ISS ≥16-added R-squared value
Adverse outcome	<0.0001	<0.0001	0.0112	<0.0001	<0.0001	<0.0001	0.30	0.30	0.32
Death	<0.0001	>0.1000	0.0032	<0.0001	>1.0000	>1.0000	0.23	0.24	0.24
Increased ICU stay	<0.0001	<0.0001	>0.1000	<0.0001	0.0005	<0.0001	0.38	0.38	0.38
Increased total LOS	<0.0001	<0.0001	>0.1000	<0.0001	0.0036	<0.0001	0.25	0.25	0.26
Not following DC commands	<0.0001	>0.1000	>0.1000	<0.0001	>0.1000	>0.1000	0.40	0.41	0.41
Ventilator	<0.0001	<0.0001	>0.1000	<0.0001	0.0003	<0.0001	0.43	0.43	0.44
Significant items	6/6	4/6	2/6	6/6	4/6	4/6	–	–	

## Discussion

Audit of fall mechanisms (2018-2020)

The 2018-2020 audit of all patients with a fall mechanism and ICH showed that most patients experienced a ground-level fall, whereas a much smaller percentage experienced a fall down multiple steps or from a height. This distribution is similar to that reported by Taylor et al. for low-energy versus high-energy falls in patients with TBI [1]. Compared with ground-level falls in the current study, falls down multiple steps or from a height had a lower proportion of patients aged ≥70 years and higher proportions of GCS 3-8, GCS 3-12, EC-ISS ≥6, WBC ≥13.0/µL, hospital stay >5 days, and need for mechanical ventilation. In contrast, Taylor et al. did not find differences in risk conditions or undesirable outcomes between low-energy and high-energy falls among patients with TBI [1]. However, it is of substantial concern that head AIS scores and brain CT findings were not reported in their manuscript [1]. The authors stated that TBI was identified using AIS codes in the registry and that TBI severity was described solely by GCS [1].

Categorizations by mechanism of injury (2018-2024)

Risks and outcomes were collated for all patients with ICH, regardless of whether the injury resulted from a ground-level fall or another blunt trauma mechanism. Compared with patients with ground-level falls, those with other blunt trauma mechanisms had a lower proportion of patients aged ≥70 years and higher proportions of GCS 3-8, GCS 3-12, ISS ≥16, EC-ISS ≥6, admission systolic blood pressure <100 mmHg, admission glucose ≥150 mg/dL, and WBC ≥13.0/µL. Patients with other blunt trauma mechanisms also had higher proportions of mortality, failure to follow commands at hospital discharge, ICU stay ≥2 days, hospital stay >5 days, adverse outcomes, and need for mechanical ventilation. Although patients with ground-level falls had lower risk conditions and better outcomes, they still accounted for a substantial proportion of those with adverse outcomes, with a total hospital stay of nearly 1,000 days per year. Among ground-level fall survivors, 50% required post-hospitalization care in acute rehabilitation, subacute nursing, or long-term acute care facilities. Thus, patients with ground-level falls and ICH represent a significant burden on trauma center operations and the post-discharge healthcare delivery system.

The only study with some similarities to our investigation is that of Lecky et al. [12]. Their study was based on a multi-center, large database of patients with TBI, categorized as having low-energy falls or other (high-energy) blunt trauma mechanisms. Mortality was similar between the two groups; however, fewer than 50% of patients had abnormal brain CT findings, and fewer than 50% were admitted to the hospital. This contrasts with the current study, in which all patients had an ICH, were admitted to the trauma center, and mortality was higher in the other blunt trauma mechanisms group compared with the ground-level fall cohort. Similar to our finding regarding the burden of ground-level falls on healthcare resources, the Lecky investigators concluded that low-energy falls are a significant contributor to the overall TBI disease burden.

WBC and glucose cutoff points for those without and with an adverse outcome

Importantly, the cutoff levels were selected because they approximated the upper 25th percentile of the WBC and glucose distributions. Other investigators have used a similar strategy to identify cutoff points for risk conditions, selecting levels that correspond to the upper 25th percentile of all values [23-25].

Univariate risk condition analyses

Six key risk conditions (GCS 3-8, EC-ISS ≥6, ICH AIS ≥4, admission systolic blood pressure <100 mmHg, glucose ≥150 mg/dL, and WBC ≥13.0/µL) each had a significant univariate association with the six undesirable outcomes (adverse outcome, hospital death, ICU stay ≥2 days, hospital stay >5 days, failure to follow commands at hospital discharge, and need for mechanical ventilation). The GCS is widely recognized as a measure of brain function that is useful for predicting outcomes [26,27]. We have previously shown in a subset of the current study population that EC-ISS ≥6, ICH AIS ≥4, and admission systolic blood pressure <100 mmHg are associated with undesirable outcomes [13].

Although admission glucose ≥150 mg/dL did not vary with age, proportions were higher in patients with other blunt trauma mechanisms, GCS 3-8, ISS ≥16, EC-ISS ≥6, ICH AIS 4-5, and admission systolic blood pressure <100 mmHg. Because admission glucose ≥150 mg/dL was not age-dependent, diabetes is unlikely to have been a substantial factor in the development of hyperglycemia [28,29]. Two studies of trauma patients reported that the prevalence of diabetes among those with hyperglycemia was only 5.1%-9.1% [14,30]. The associations with other risk conditions suggest that hyperglycemia was related to stress responses associated with the severity of traumatic injury.

Proportions of admission WBC ≥13.0/µL were higher in patients with other blunt trauma mechanisms, GCS 3-8, ISS ≥16, EC-ISS ≥6, ICH AIS 4-5, and admission systolic blood pressure <100 mmHg than in the corresponding counterpart categories. These findings indicate that leukocytosis is a stress-induced response related to injury severity. WBC and glucose levels were significantly positively correlated, and proportions of glucose ≥150 mg/dL were higher in patients with WBC ≥13.0/µL than in those without leukocytosis. Together, these observations support the notion that hyperglycemia and leukocytosis reflect the patient’s level of injury severity.

A surge in systemic catecholamines is associated with stress-induced hyperglycemia [31-33] and stress-induced leukocytosis [34,35]. Additionally, increased systemic catecholamine levels have been observed in patients with TBI [36-38], non-brain-injured blunt trauma [39], and major burn injury [40]. Another study reported that catecholamine levels progressively increase as GCS decreases [41]. Stress hyperglycemia in patients with TBI is more severe in those with undesirable outcomes (hospital mortality and increased injury severity) than in those with desirable outcomes [30,42,43]. Similarly, in patients with TBI, stress leukocytosis has been associated with undesirable outcomes [17,18].

We have identified evidence that may explain the frequent occurrence of hyperglycemia and leukocytosis in patients without adverse outcomes. Acute psychological trauma is common among individuals experiencing interpersonal violence [44]. Another study found that, among patients evaluated in the trauma room of a level-one trauma center, an acute psychological disorder was documented in 20% of cases, even though ISS was <16 in 85% of patients [45]. Moreover, acute psychological trauma has been shown to increase catecholamine [46] and cortisol [47] levels. In a study of trauma patients, catecholamine levels were elevated in patients with both increased and low ISS relative to laboratory control values, but levels were higher in patients with increased ISS [48]. The authors concluded that emotional trauma was likely an inciting factor [48]. These observations suggest that stress-induced hyperglycemia and leukocytosis may be related to psychological trauma as well as physical injury.

Risk score and adverse outcome analysis

Using the six key risk conditions (GCS 3-8, EC-ISS ≥6, ICH AIS ≥4, admission systolic blood pressure <100 mmHg, glucose level ≥150 mg/dL, and WBC ≥13.0/µL), a risk score for an adverse outcome was computed and analyzed. The AUC was clinically important and indicated that the risk score elements, in aggregate, could be used to predict the occurrence of an adverse outcome. The dichotomized representation of the risk score provided a substantial OR and RR.

Multivariate risk condition analyses with undesirable outcomes

Six risk conditions (decreased GCS, increased EC-ISS, admission systolic blood pressure <100 mmHg, increased ICH AIS, glucose level ≥150 mg/dL, and WBC ≥13.0/µL) were simultaneously assessed using multivariate analysis for each undesirable outcome (an adverse outcome, death, an increased ICU stay, an increased hospital stay, not following commands at hospital discharge, and a need for mechanical ventilation). The R-squared values for the six models ranged from 0.23 to 0.43, indicating their clinical relevance [49]. Across the six undesirable outcomes, decreased GCS and increased ICH AIS were independently associated with all six outcomes; increased EC-ISS, glucose ≥150 mg/dL, and WBC ≥13.0/µL were independently associated with four outcomes; and admission systolic blood pressure <100 mmHg was independently associated with two outcomes. These analyses provide further supportive evidence for the utility of these six risk conditions in predicting undesirable outcomes.

The fact that the non-GCS risk conditions remain independently associated with outcomes alongside GCS underscores their validity and clinical importance. When another blunt trauma mechanism (yes/no) or ISS ≥16 (yes/no) was added separately to the six other potential independent variables, the resulting increase in R-squared for each model was negligible. The lack of additional predictive value from ISS ≥16 further supports the significance of the other risk conditions, given that ISS is a recognized predictor of undesirable outcomes [50-52]. It is important to note that the risk conditions collectively represent a composite profile of a blunt trauma patient with intracranial hemorrhage, encompassing diverse physiologic and anatomic domains. These components are as follows: GCS as a measure of brain function; ICH AIS as an indicator of intracranial hemorrhage volume; EC-ISS as a measure of extracranial anatomic injury; hypotension as an indicator of circulatory instability; and WBC and glucose level as measures of catecholamine responses.

Limitations of the study

The main limitation of the current study is its retrospective design, which precludes definitive conclusions regarding causality. The use of partial years (2018-2020) and single-center data may limit generalizability and introduce selection bias. Because a modest proportion of patients in the current study were managed at the trauma center during the COVID-19 Ohio stay-at-home order, the pandemic may have influenced the types of patients admitted. Additionally, preinjury medications and medical conditions can affect blood glucose, white blood cell count, cortisol, and catecholamine levels, and their assessment would have provided a more comprehensive understanding of these physiologic responses.

## Conclusions

Compared with patients experiencing a ground-level fall, those with other blunt trauma mechanisms exhibit worse risk profiles and poorer outcomes among patients with ICH. Stress-induced leukocytosis and hyperglycemia were frequently associated with multiple adverse outcomes. Future investigators should consider incorporating stress leukocytosis and hyperglycemia as indicators of risk conditions that likely stem from catecholamine responses after trauma. Future research is necessary to confirm the observations in the current study.
